# Comparison of White Matter Structure of Drug-Naïve Patients With Bipolar Disorder and Major Depressive Disorder Using Diffusion Tensor Tractography

**DOI:** 10.3389/fpsyt.2021.714502

**Published:** 2022-02-14

**Authors:** Akihiro Koreki, Richi Niida, Akira Niida, Bun Yamagata, Sachiko Anamizu, Masaru Mimura

**Affiliations:** ^1^Department of Neuropsychiatry, Keio University School of Medicine, Tokyo, Japan; ^2^Department of Psychiatry, National Hospital Organization Shimofusa Psychiatric Medical Center, Chiba, Japan; ^3^Department of Radiology, Tomishiro Central Hospital, Tomigusuku, Japan

**Keywords:** bipolar disorder, major depressive disorder, diffusion tensor tractography, white matter, anterior thalamic radiation

## Abstract

**Background:**

The presence of microstructural white matter (WM) abnormalities in individuals with bipolar disorder (BD) has previously been reported. However, the interpretation of data is challenging because pharmacological treatment has a potential effect on WM integrity. To date, no study has compared the differences in WM structure among drug-naïve BD patients, drug-naïve major depression disorder (MDD) patients, and healthy controls (HC) using the visual evaluation method of diffusion tensor tractography (DTT).

**Methods:**

This retrospective study included 12 drug-naïve patients with BD, 15 drug-naïve patients with MDD, and 27 age- and sex-matched HC individuals. Visual evaluation, fractional anisotropy (FA), and apparent diffusion coefficient (ADC) were analysed in the anterior thalamic radiation (ATR) as a tract of interest using the optimal follow-up truncation threshold. They were also analysed in the cingulate fasciculus, superior longitudinal fasciculus, inferior longitudinal fasciculus, inferior fronto-occipital fasciculus, uncinate fasciculus, and fornix.

**Results:**

No significant differences were found in the FA or ADC of any tract. However, visual evaluation revealed poorer depiction of ATR in patients with BD than in patients with MDD and HC individuals (*p* = 0.004). Our *post-hoc* analysis showed a significant difference between BD and HC patients (*p* = 0.018).

**Conclusions:**

The visual evaluation method of DTT revealed poor depiction of ATR in patients with BD compared with HC individuals and MDD patients, suggesting microstructural WM abnormalities of ATR in BD.

## Introduction

The difficulty of diagnosing bipolar disorder (BD) in clinical practise resides in the need to identify a specific brain abnormality. Disruptions in the cerebral white matter (WM) integrity are considered a biological indicator of BD. Previous studies have investigated the effectiveness of diffusion tensor imaging (DTI) in the identification of this abnormality (1–3). However, the results are still controversial regarding the clinical applicability of DTI in the differentiation between BD and major depressive disorder (MDD) (4, 5).

Compared to DTI, diffusion tensor tractography (DTT) is considered more powerful due to several evaluation features including visual evaluation, fractional anisotropy (FA), and apparent diffusion coefficient (ADC). Previous studies have utilised FA and ADC. However, inconsistent results were obtained, possibly due to the averaging of values with the surrounding voxels resulting in a weaker effect (6) or due to the reduction in FA caused by the partial volume effect of the cerebral spinal fluid (7). Therefore, the assessment of microstructural WM abnormalities is insufficient. However, visual evaluation methods can overcome this limitation (8, 9). Our previous DTT study has indicated the potential usefulness of the visual evaluation of WM integrity for the distinction of BD patients from healthy controls (HC), which revealed poor depiction of the anterior thalamic radiation (ATR) in BD (9).

Additionally, difficulties in eliminating drug effects lead to challenges in imaging studies of BD. In most studies, patients with BD and MDD are under treatment. Several studies have found that pharmacological treatment has a potential effect on WM integrity (10–12), leading to difficulties in interpretation. Therefore, in the present study, we investigated the effectiveness of several DTT evaluation methods (visual evaluation, FA, and ADC) among drug-naïve BD patients, drug-naïve MDD patients, and HC. Our study focused on the evaluation of the following seven major WM tracts, based on previous studies: the ATR (13, 14); the cingulate fasciculus (CF) (15–17); the superior longitudinal fasciculus (SLF) [Amelia (18)]; the inferior longitudinal fasciculus (ILF) (19, 20); the inferior fronto-occipital fasciculus (IFOF) (19); the uncinate fasciculus (UF) (19); and the fornix (FX) (21, 22). Our prior studies suggested that poor depiction of ATR may be a potential biological marker of BD. Indeed, a recent meta-analysis of patients with BD has shown that aberrant ATR is a disease marker (23). In addition, ATR is considered to play a role in mood regulation, particularly for negative feelings such as sadness, and also to regulate positive feelings through a functional interplay with the medial forebrain bundle (24, 25).

Based on these previous findings, we formulated the hypothesis that ATR has a poor depiction in BD patients compared with HC (9), and that ATR is preserved in individuals with MDD compared with HC (8). Therefore, in the present study, we compared the seven aforementioned WM structures, especially ATR, among drug-naïve BD patients, drug-naïve MDD patients, and HC, using the visual evaluation method of DTT as well as FA and ADC.

## Methods

### Participants

This study included a total of 54 participants: 12 individuals with BD, 15 with MDD, and 27 age- and sex-matched HC. The diagnosis was made by psychiatrists according to the Structured Clinical Interview for the Diagnostic and Statistical Manual of Mental Disorders IV Edition Text Revision (SCID) of DSM-IV-TR (26). The following characteristics excluded patients from the study: history of other neurological disorders or head injury; pre-existing dementia; substance dependence; and contraindications for magnetic resonance imaging (MRI), including pacemaker use. All individuals with BD and MDD were drug-naïve and in a depressive state at the time of the MRI scan. Some patients were referred from other departments such as the orthopaedic surgery and gynaecology and took pain medications and herbal medicines as needed, but we confirmed none of the patients took antipsychotic medication. The participants were instructed to complete the Hamilton Depression Rating Scale (HAM-D) questionnaire (27). HC status was assessed through a structured interview to confirm right-handedness, absence of psychiatric history including schizophrenia or mood disorders, absence of comorbid conditions including hypertension or diabetes mellitus, and absence of family history of psychiatric illnesses. Written informed consent was obtained from all participants after offering a detailed explication of the study. This study was approved by the Ethics Committee of the Tomishiro Central Hospital (former Nanbu Hospital, H29R036).

### Data Acquisition

We performed the brain MRIs using a 1.5-T MRI device (Achieva Nova; Koninklijke Philips Electronics NV, Amsterdam, The Netherlands) with an 8-channel sensitivity encoding (SENSE)-head coil. Diffusion-weighted data were collected with a spin-echo single-shot echo-planar imaging sequence (repetition time/echo time/flip angle: 6,231 ms/75 ms/90°) with a SENSE parallel-imaging scheme (reduction factor: 2.0). Diffusion gradients were applied in 15 spatial directions, with b-values of 0 s/mm^2^ and 800 s/mm^2^. Images were obtained using a 116 × 116 matrix and a 230 × 230-mm field of view zero filled to 256 × 256 pixels. The reconstruction voxel size was 1.75 × 1.75 × 1.75 mm. Fifty transverse sections of 3-mm slice thickness were acquired. Two measurements were obtained and averaged. The total acquisition time was 3 min and 26 s.

**Figure 1:**
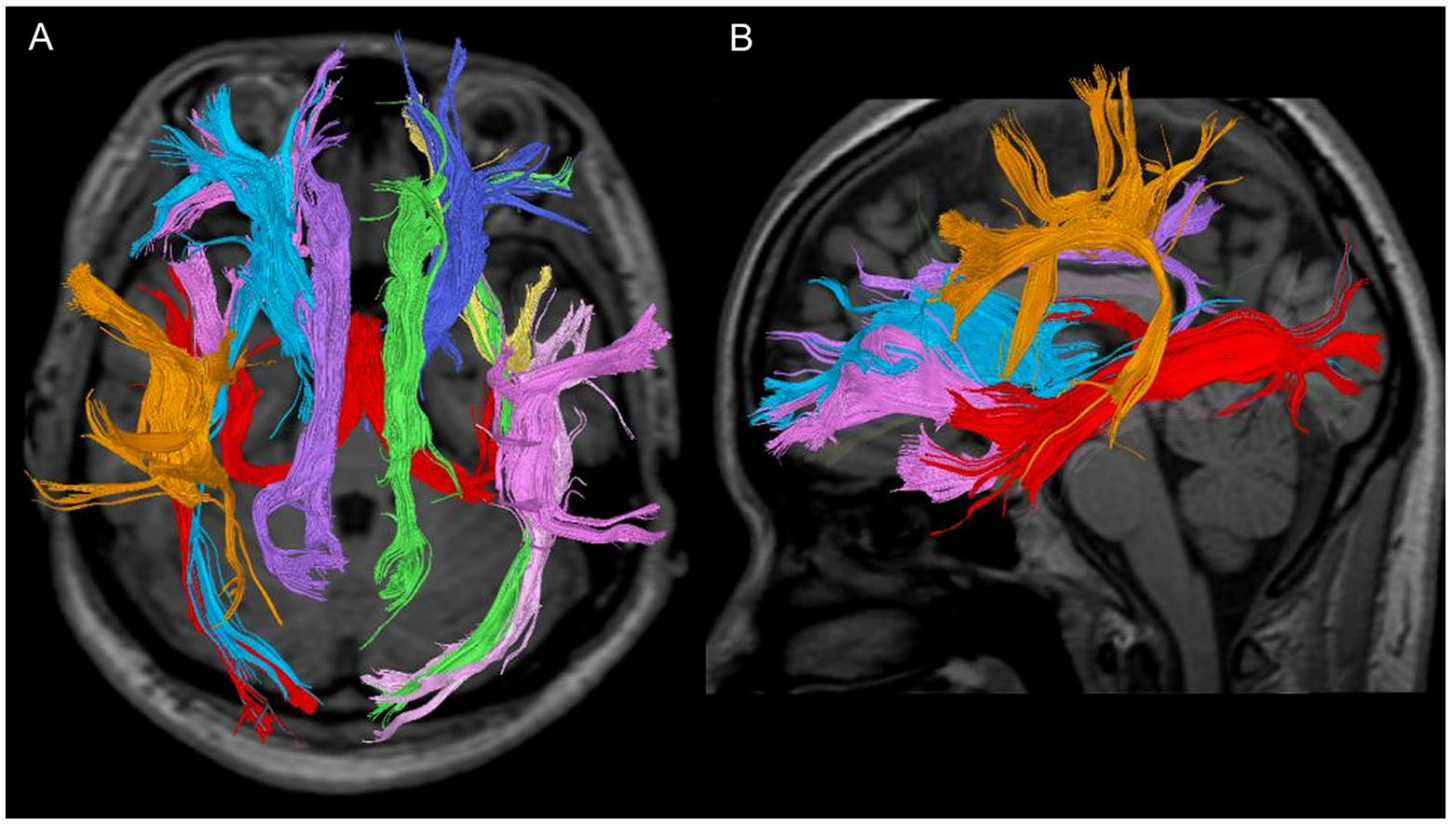
Seven fibre bundles in a 28-year-old healthy woman. **(A)** Tractography with a trans-axial image. **(B)** Tractography with a weighted sagittal image.

**Figure 2:**
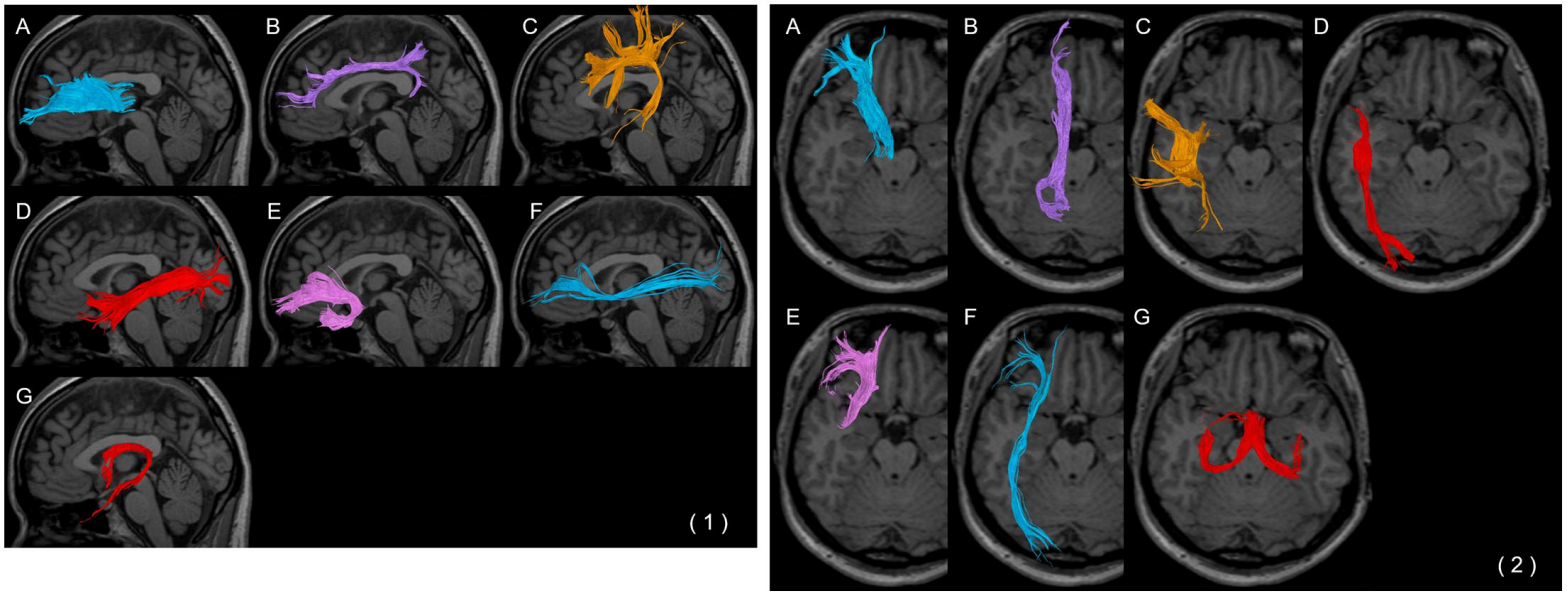
Each fibre bundle with a (1) weighted sagittal image and (2) trans-axial image. **(A)** Anterior thalamic radiation, **(B)** cingulate fasciculus, **(C)** superior longitudinal fasciculus, **(D)** inferior longitudinal fasciculus, **(E)** uncinate fasciculus, **(F)** inferior fronto-occipital fasciculus, and **(G)** fornix.

The DTT method can be used to identify macroscopic nerve fascicle distribution by determining the continuousness of the diffusion anisotropy vectors of adjacent voxels based on the DTI. The collected DTI data were sent to a workstation of Philips Extended MR WorkSpace (release 2.6.3.2) and analysed using FiberTrak software (release 2.6.3.2; Koninklijke Philips Electronics NV, Amsterdam, The Netherlands). The step width of fibre tracking was set at 10 mm as default, and the conditions for the discontinuation of fibre tracking were an FA value of 0.1–0.35 and a flip angle of 27°. By using the multiple regions of interest (ROI) approach, INCLUDE-ROIs, wherein included regions were specified, were manually set with the tractography atlas as the reference. On the other hand, EXCLUDE-ROIs, wherein excluding regions were specified, were set in regions containing other mixed nerve fibres to exclude these fibres (28, 29). For example, the corticospinal and corticopontine tracts are mixed in the ATR, but pure ATR can be visualised by setting EXCLUDE-ROIs, including the midbrain tracts. Additionally, the EXCLUDE-ROIs were set between the cerebral hemispheres to visualise pure nerve fascicles and exclude anatomically consistent nerve fibres that appeared continuous to the contralateral cerebral hemisphere due to decussation, such as in the corpus callosum (8). Likewise, the bilateral ATR, CF, SLF, ILF, UF, IFOF, and FX were depicted (Figures 1, 2). The mean FA and ADC values of voxels containing each nerve fascicle were determined. To ensure sufficient data quality for reliable tractography at 1.5T resolution, we performed imaging of a 35-year-old man (healthy volunteer) for 10 consecutive days using the same scanning equipment, DTT software application, and device operator as a preliminary investigation. As a result of this preliminary study, 10 comparably favourable ATR depictions were acquired.

### Visual Evaluation of DTT

Visual evaluation of DTT is a useful tool in the detection of WM microstructure abnormalities in individuals with psychiatric illnesses, including BD and dementia (8, 9). In this study, two board-certified radiology specialists (RN, AN) determined poor depiction of the tracts.

**Figure 3:**
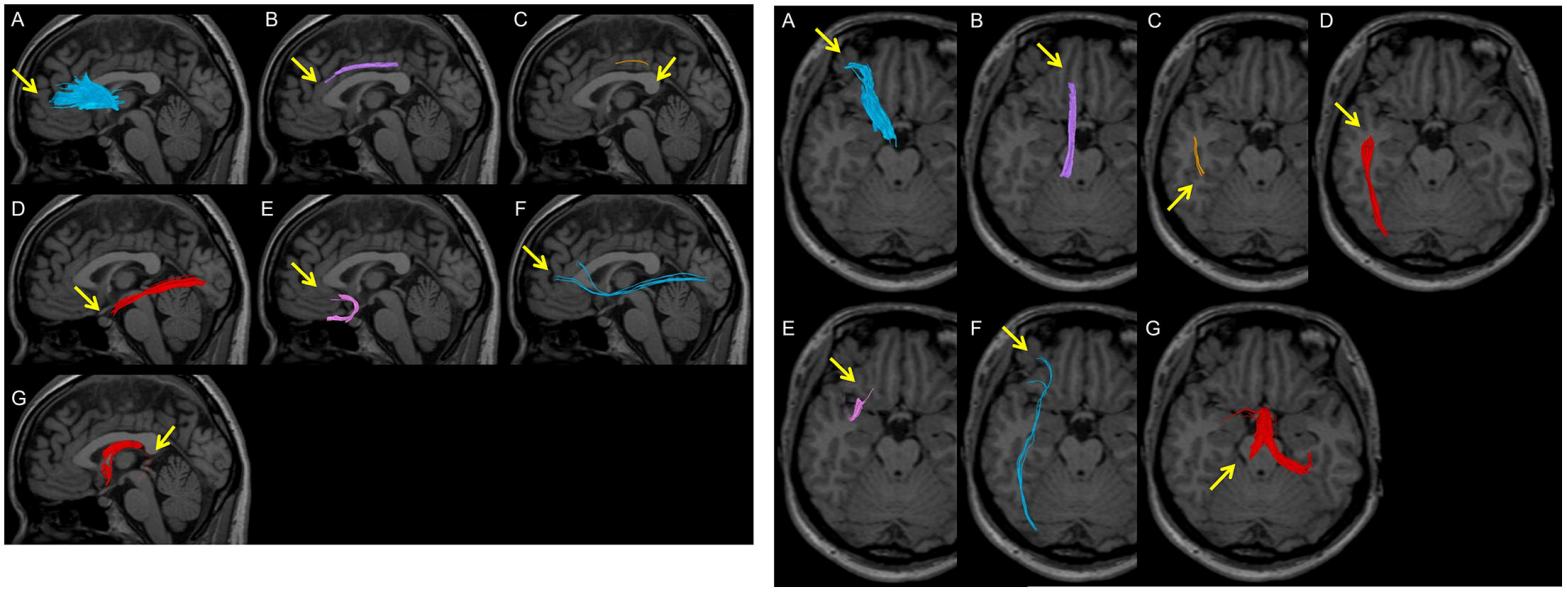
Examples of poor depiction in visual evaluation. Arrows indicate poor depictions. **(A)** A peripheral fibre bundle of the anterior thalamic radiation from the middle between the brain surface and the genu of the corpus callosum is not depicted. **(B)** The anterior part of the cingulate fasciculus is not depicted in the superior area of the corpus callosum. **(C)** A part of the superior longitudinal fasciculus between the parietal and temporal regions is not depicted. **(D)** A part of the inferior longitudinal fasciculus between the temporal pole and the occipital pole is not depicted. **(E)** A part of the uncinate fasciculus in the occipitofrontal area is not depicted. **(F)** A thin fibre bundle of the inferior fronto-occipital fasciculus is observed and is not completely depicted between the frontal area and the occipital pole. **(G)** The crus of the fornix is not depicted.

ATR was assessed in both sagittal and axial sections; poor depiction was defined as the failure of at least one ATR fibre in reaching the boundary between the grey and the white matter. CF was assessed in the sagittal section; poor depiction was defined as the failure of at least one CF fibre bundle to reach the genus of the corpus callosum. SLF was assessed in the sagittal section; poor depiction was defined as the failure to observe at least one SLF fibre between the parietal and temporal regions. ILF was assessed in both sagittal and axial sections; poor depiction was defined as the failure to observe at least one ILF fibre bundle between the subcortical regions of the temporal and the occipital pole. UF was assessed in the sagittal section; poor depiction was defined as the failure of at least one UF in the orbitofrontal regions to reach the genus of the corpus callosum. IFOF was assessed in both sagittal and axial sections; poor depiction was defined as the failure of at least one IFOF from the frontal subcortical region to reach the subcortical region of the occipital pole. A poor depiction of FX was defined as the non-tracking of at least one fibre bundle of the crus fornicis (Figure 3).

Regarding the DTT software parameters, the default threshold for fibre tracking was set to be extremely low for maximising the power of fibre tracking, leading to the underestimation of the microstructural change in patients. Therefore, to set the optimal threshold for fibre tracking, the FA value necessary to visualise fibres in each HC was identified, with the subsequent evaluation of the maximum FA value necessary to visualise fibres in all HC, except in cases where the value was 0, which was defined as the “optimal follow-up truncation threshold.” Finally, FA, ADC, and good/poor depiction (in accordance with the aforementioned definition of poor depiction) were assessed. We confirmed the reliability of this evaluation in the same participants (data not shown).

### Statistical Analysis

One-way analysis of variance was used to compare age, FA, and ADC. Fisher's exact-test was used to compare the sex ratio and the ratio of good/poor visualisation. The Holm method was used for *post-hoc* analysis. A student's *t*-test was used to compare the HAM-D and duration of illness (DOI) between BD and MDD. Statistical significance was set at *P* < 0.05. *R* (4.02) was used for statistical data processing.

## Results

### Patient Characteristics

Among the 12 individuals with BD, there were five male and seven female patients with a mean age of 33.8 ± 7.9 years and a mean DOI of 5.7 ± 4.0. Among the 15 individuals with MDD, there were three male and 12 female patients with a mean age of 34.6 ± 5.4 years and a mean DOI of 3.4 ± 1.6. Among the 27 age- and sex-matched HC, there were eight male and 19 female individuals with a mean age of 34.7 ± 6.8 years. The DOI in the BD and MDD groups was relatively long because, before being referred to our psychiatric department, the patients were followed up at other departments. The scores on the HAM-D for the BD and MDD groups were 20.8 ± 4.0 and 19.7 ± 3.7, respectively (Table 1). No significant difference was found regarding sex and age between the three groups. Additionally, no significant difference was found in the DOI or HAM-D in the BD and MDD groups.

**Table 1:** Clinical characteristics of the total sample (*n* = 54).

	**HC**	**BD**	**MDD**	***P*-value**
*N*	27	12	15	
Age, y (mean ± SD)	34.7 ± 6.8	33.8 ± 7.9	34.6 ± 5.4	0.93
Gender (male/female)	8/19	5/7	3/12	0.50
DOI, y (mean ± SD)	–	5.7 ± 4.0	3.4 ± 1.6	0.053
HAMD	–	20.8 ± 4.0	19.7 ± 3.7	0.47

*HC, healthy control; BD, bipolar disorder; MDD, major depressive disorder; DOI, duration of illnesses; HAMD, Hamilton's Rating Scale for Depression*.

### Imaging Analysis

**Figure 4:**
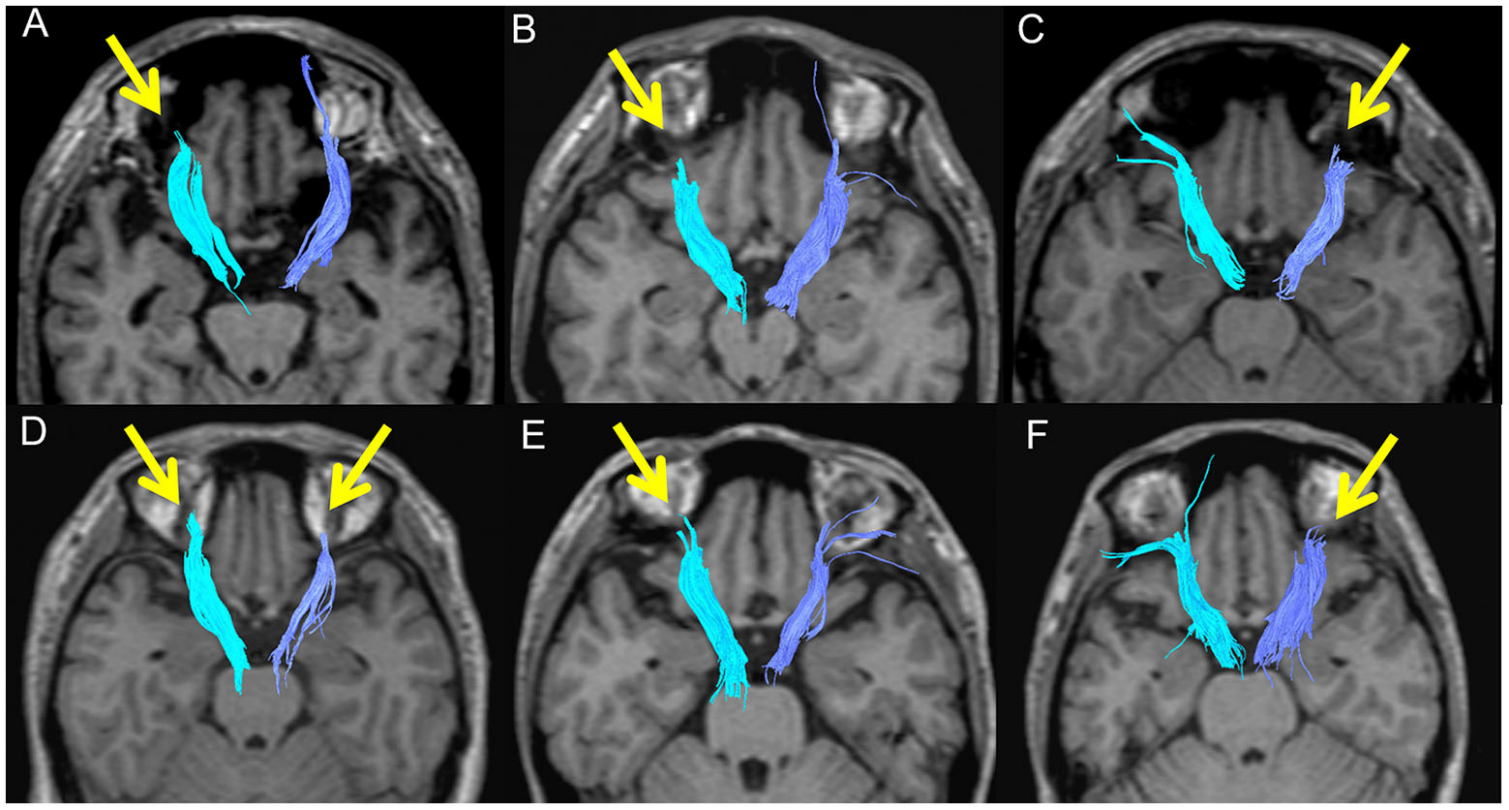
Poor depictions of the anterior thalamic radiation in patients with bipolar disorder (**A**: 46M, **B**: 38M, **C**: 26M, **D**: 38F) and major depressive disorder (**E**: 37F, **F**: 39F).

The FA and ADC values of the nerve fascicles are presented in Table 2. No significant difference was found regarding the FA and ADC values of any tract between the three groups. The number of samples with poor depiction of each WM fibre bundle using the optimal follow-up censoring FA threshold are shown in Table 3. A significant difference was found between the ATRs of the three groups in the visual evaluation using the optimal follow-up censoring FA threshold (*P* = 0.004). A *post-hoc* analysis revealed that the poor depiction of ATR was more frequently observed in the BD group than in the HC group (*P* = 0.018). However, no significant difference was observed between the BD and MDD groups, nor between the MDD and HC groups. Poor depictions of the anterior thalamic radiation in patients with bipolar disorder and major depressive disorder were shown in Figure 4. Additionally, a significant difference was found in the CF (*P* = 0.009) of the three groups in the visual evaluation using the optimal follow-up censoring FA threshold. However, *post-hoc* analysis revealed no significant difference (e.g., *P* = 0.07 between BD and HC). No significant differences were observed in other nerve fibre bundles.

**Table 2:** The FA and ADC values (SD) of the nerve fascicles.

**Bundle**	**Site**	**HC (*n* = 27)**	**BD (*n* = 12)**	**MDD (*n* = 15)**	
		**FA**			***P***
ATR	R/L	0.464 (0.065)/0.454 (0.059)	0.463 (0.013)/0.474 (0.016)	0.455 (0.019)/0.471 (0.030)	0.846/0.271
CF	R/L	0.500 (0.024)/0.536 (0.016)	0.506 (0.022)/0.494 (0.157)	0.500 (0.013)/0.540 (0.018)	0.679/0.213
SLF	R/L	0.450 (0.019)/0.512 (0.020)	0.453 (0.018)/0.513 (0.023)	0.452 (0.018)/0.509 (0.017)	0.851/0.848
ILF	R/L	0.491 (0.031)/0.505 (0.017)	0.483 (0.018)/0.501 (0.024)	0.481 (0.016)/0.501 (0.018)	0.424/0.728
UF	R/L	0.440 (0.022)/0.452 (0.031)	0.443 (0.020)/0.457 (0.018)	0.442 (0.011)/0.460 (0.023)	0.860/0.619
IFOF	R/L	0.502 (0.020)/0.510 (0.028)	0.508 (0.028)/0.463 (0.149)	0.499 (0.031)/0.508 (0.025)	0.614/0.169
FX	–	0.362 (0.016)	0.352 (0.038)	0.358 (0.018)	0.433
		**ADC**			***P***
ATR	R/L	0.786 (0.023)/0.780 (0.023)	0.788 (0.025)/0.783 (0.017)	0,788 (0.022)/0.773 (0.032)	0.970/0.569
CF	R/L	0.802 (0.032)/0.797 (0.026)	0.805 (0.025)/0.740 (0.235)	0.802 (0.026)/0.795 (0.025)	0.961/0.314
SLF	R/L	0.790 (0.040)/0.77 0 (0.024)	0.789 (0.020)/0.776 (0.022)	0.785 (0.030)/0.767 (0.019)	0.885/0.543
ILF	R/L	0.821 (0.046)/0.823 (0.030)	0.831 (0.046)/0.834 (0.032)	0.817 (0.020)/0.823 (0.023)	0.513/0.497
UF	R/L	0.821 (0.027)/0.819 (0.029)	0.825 (0.021)/0.827 (0.020)	0.826 (0.021)/0.814 (0.026)	0.808/0.443
IFOF	R/L	0.825 (0.030)/0.815 (0.025)	0.836 (0.041)/0.758 (0.240)	0.814 (0.019)/0.819 (0.028)	0.185/0.297
FX	–	1.508 (0.175)	1.610 (0.340)	1.499 (0.181)	0.355

*HC, healthy control; BD, bipolar disorder; MDD, major depressive disorder; FA, fractional anisotropy; ADC, apparent diffusion coefficient; ATR, anterior thalamic radiation; CF, cingulate fasciculus; SLF, superior longitudinal fasciculus; ILF, inferior longitudinal fasciculus; UF, uncinate fasciculus; IFOF, inferior fronto-occipital fasciculus; FX, fornix; R, right; L, left; SD, standard deviation*.

**Table 3:** Poor depiction using both default and optimal follow-up censoring FA threshold.

**Bundle**	**Optimal threshold (R/L)**	**HC (*n* = 27)**	**BD (*n* = 12)**	**MDD (*n* = 15)**	***P***	
ATR	0.24/0.25	0	4 (2/3)	2 (1/1)	**0.004**	**BD>HC**
CF	0.30/0.35	0	3 (2/1)	0	**0.009**	ns
SLF	0.21/0.33	7 (5/2)	3 (1/2)	3 (1/2)	ns	–
ILF	0.23/0.28	1 (1)	1 (1)	0	ns	–
UF	0.25/0.26	4 (1/3)	3 (1/2)	2 (2)	ns	–
IFOF	0.22/0.20	3 (2/1)	3 (2/1)	4 (2/3)	ns	–
FX	0.1	4	1	2	ns	–

*N of poor depiction of either (right/left) bundle was described*.

*HC, healthy control; BD, bipolar disorder; MDD, major depressive disorder; FA, fractional anisotropy; ATR, anterior thalamic radiation; CF, cingulate fasciculus; SLF, superior longitudinal fasciculus; ILF, inferior longitudinal fasciculus; UF, uncinate fasciculus; IFOF, inferior fronto-occipital fasciculus; FX, fornix; R, right; L left; ns, not significant*.

*The bold values mean significant P values*.

## Discussion

To our knowledge, this is the first study to demonstrate the effectiveness of the poor depiction of ATR in visual evaluation as a biological marker in drug-naïve individuals with BD compared with both drug-naïve MDD patients and HC individuals. The current study supports our previous finding that patients with BD undergoing pharmacological treatments showed abnormalities in ATR (9), which is consistent with other previous studies (23, 25, 30, 32). Considering the depressive state of all individuals during the MRI scan, the poor depiction of ATR may suggest a pathophysiological mechanism involving ATR abnormalities in individuals with BD. ATR is a WM fibre bundle which serves as a connexion between the prefrontal lobe (mainly the dorsolateral prefrontal cortex) and the thalamus through the anterior limb of the internal capsule. Additionally, ATR has projections to the limbic system. ATR plays a role in mood regulation, particularly for negative feelings such as sadness. Additionally, ATR may also regulate positive feelings through a functional interplay with the medial forebrain bundle (24, 25). Therefore, ATR dysfunction may lead to dysfunctions in mood regulation, manifesting as mood swings, in individuals with BD. Abnormalities in ATR have been frequently observed in individuals with BD (9, 23, 25, 30). A recent meta-analysis of patients with BD reported several aberrant WM microstructures, including in ATR, in individuals at risk of BD. Additionally, a greater FA decrease was observed in the ATR of individuals with BD, compared with those only at risk of BD, suggesting that aberrant ATR is a disease marker (23).

The abnormalities in ATR observed in drug-naïve individuals with BD may serve as a potential treatment target. To date, no randomised controlled study has been conducting to explore the drug effects on WM in individuals with BD. However, previous studies have demonstrated a relationship between WM changes and pharmacological treatments for BD. The specific WM regions associated with treatments for BD remain controversial (10, 12). A recent meta-analysis of BD studies showed a positive effect of lithium on FA in some regions, including ATR (23). An inverse relationship was indicated between the proportion of patients with BD taking lithium and the FA changes of the WM in the bilateral ATR, corpus callosum, SLF, ILF, and corticospinal tract. Additionally, the meta-analysis reported a positive antipsychotic effect on FA in other regions of the WM (i.e., the ILF arcuate fasciculus, cingulum, anterior corona radiata, and interstriatal WM). However, specific WM changes closely related to treatment responses remain unknown; hence, further study is required.

In the present study, only the visual evaluation method, not FA and ADC, revealed ATR abnormalities. One possible explanation is that BD-specific nerve fibre impairments tend to be localised rather than occurring throughout the nerve fibre. Another explanation is that the peripheral part of ATR may be slightly rough, which would lead to the poor depiction but does not influence the FA value. In the FA and ADC values, the ROI method was mainly used, and these values were averaged with the surrounding voxels, which may have masked the presence of any important difference (6). The underestimation may be attributed to the reduction in FA caused by the partial volume effect exerted by the cerebral spinal fluid (7). Furthermore, in the present study, the threshold for fibre tracking was adjusted to improve sensitivity. The default threshold of the software was set to maximise the power of fibre tracking, which led to an underestimation of microstructural changes in patients. The threshold was determined based on the values in the HC group, allowing the improvement of sensitivity to detect microstructural changes.

While a significant difference in ATR was found between the BD and the HC groups, no significant difference was observed between the BD and MDD groups in the *post-hoc* analysis despite the higher prevalence of ATR abnormalities in individuals with BD. We believe that this was due to the small sample size. Our previous study, wherein all BD patients were receiving treatment, revealed ATR abnormalities unlike those in HC individuals (9). Another study showing ATR abnormalities in patients with Alzheimer's disease compared with MDD patients and HC individuals suggests the preservation of ATR in MDD (8). These findings highlight the specificity of ATR abnormalities in BD compared with other mood disorders. Moreover, a significant difference was found in the CF of the three groups. However, *post-hoc* analyses showed not significant differences, which may be attributed to the small sample size. Further studies are required to confirm this finding.

The present study has several strengths. First, the participants were limited to drug-naïve individuals with BD and MDD, which allowed an easier interpretation of the results without the influence of pharmacological treatment. Second, visual evaluation is more easily applicable in clinical practise compared with FA and ADC; this is due to the non-applicability of the FA and ADC group study results to individuals. Third, we used the optimal follow-up truncation threshold, leading to an improvement in the sensitivity for detecting ATR abnormalities.

Conversely, the study has several limitations. First, there may be variations in the results of the DTT analysis depending on the MRI equipment or the analysis software used. Therefore, there may be limitations in the applicability of our findings to other institutions. Additionally, this variability presents difficulties in conducting multicentre comparative evaluations. Advancements in the development of a standardised tractography method using an automated analysis software may increase the feasibility of multicentre comparative studies and the precision of differential diagnosis. Recently, diffusion kurtosis imaging and neurite orientation dispersion and density imaging have been developed, and clinical applications are expected in the near future. However, DTT is currently available and can provide useful clinical imaging information. Second, the small sample size limited the evaluation of the laterality of DTT abnormalities. However, in this and our previous study (9), and in some existing studies (25, 30), poor depiction of ATR was observed on both sides. Nonetheless, inconsistencies remain regarding the laterality of ATR abnormality (31). Further studies are therefore required to confirm these observations. Third, the resolution of 1.5T MRI is lower than that of 3T MRI, potentially leading to low sensitivity for detecting abnormalities. However, we have ensured reproducibility of our results in our preliminary study, and we also believe that this approach is more clinically applicable because 1.5T MRI is still mainly used in hospitals, not in research institutes.

## Conclusion

Visual evaluation of DTT revealed poor depiction of ATR in drug-naïve BD patients compared with HC individuals and MDD patients, suggesting microstructural changes of ATR in individuals with BD. Our findings may reveal specific abnormalities in the complex neuroanatomical background of individuals with BD, whose vulnerability may lead to the development of symptoms.

## Data Availability Statement

The raw data supporting the conclusions of this article will be made available by the authors, without undue reservation.

## Ethics Statement

The studies involving human participants were reviewed and approved by Tomishiro Central Hospital (H29R036). The patients/participants provided their written informed consent to participate in this study.

## Author Contributions

RN and AN designed and collected the study. AK and RN analysed the data. AK and RN wrote the initial draft of the manuscript and AN, BY, SA, and MM critically reviewed it. All authors contributed to data interpretation and approved the final version of the manuscript.

## Funding

This research was supported by a Grant-in-Aid for Scientific Research from Japan Society for the Promotion of Science (No. JP20H00108) and Japan Agency for Medical Research and Development (No. JP21dm0307102h0003).

## Conflict of Interest

The authors declare that the research was conducted in the absence of any commercial or financial relationships that could be construed as a potential conflict of interest.

## Publisher's Note

All claims expressed in this article are solely those of the authors and do not necessarily represent those of their affiliated organizations, or those of the publisher, the editors and the reviewers. Any product that may be evaluated in this article, or claim that may be made by its manufacturer, is not guaranteed or endorsed by the publisher.
